# NMN protects vascular endothelial cells from M1 macrophage-derived IL-1β-induced hyperpermeability by inhibiting VE-cadherin degradation

**DOI:** 10.3389/fcvm.2026.1748872

**Published:** 2026-05-18

**Authors:** Takeshi Katayoshi, Takahisa Nakajo, Natsuko Kitajima, Wakana Naka, Yuki Kamei, Taiki Fushimi, Masakatsu Kageyama, Mitsugu Akagawa, Kentaro Tsuji

**Affiliations:** 1 DHC Corporation Laboratories, Division 2, Chiba, Japan; 2 Department of Food Science, Institute of Biomedical Sciences, Tokushima University Graduate School, Tokushima, Japan

**Keywords:** hyperpermeability, interleukin-1β (IL-1β), m1 macrophages, NMN (Nicotinamide mononucleotide), vascular endothelial cells, VE-cadherin degradation

## Abstract

**Background and aims:**

Vascular endothelial (VE) dysfunction, particularly endothelial hyperpermeability, is a critical pathological process in various inflammatory vascular diseases, including atherosclerosis, vasculitis, and sepsis. Nicotinamide mononucleotide (NMN), an NAD^+^ precursor, has shown anti-inflammatory and vascular protective effects in preclinical models. However, the mechanisms by which NMN preserves endothelial barrier integrity against macrophage-derived inflammatory stimuli remain unclear. This study examined the potential protective role of NMN in endothelial hyperpermeability induced by pro-inflammatory macrophages.

**Methods:**

A three-dimensional co-culture model of human umbilical vein endothelial cells (HUVECs) and M1 macrophages was constructed to reproduce inflammatory vascular microenvironments. Endothelial permeability was evaluated by measuring fluorescently labelled dextran and LDL passages from the luminal (top) to the abluminal side (bottom) of the insert.

**Results:**

M1 macrophage co-culture increased HUVEC permeability, and NMN pretreatment attenuated this hyperpermeability. Mechanistic analysis revealed that interleukin-1β (IL-1β) released by M1 macrophages was the primary contributor to endothelial hyperpermeability. NMN suppressed IL-1β-induced cell-cell gap formation and VE-cadherin degradation in HUVECs by inhibiting nuclear factor kappa B (NF-*κ*B) pathway activation. These findings indicate that NMN prevents IL-1β-induced NF-*κ*B activation and subsequent VE-cadherin degradation, thereby protecting against endothelial hyperpermeability caused by intercellular gap formation. Other NAD^+^ precursors, including nicotinamide riboside, similarly protected against IL-1β-induced hyperpermeability and VE-cadherin degradation. Alternatively, the NAD^+^-dependent deacetylase sirtuin 1 (SIRT1) inhibitor EX527 or SIRT1 siRNA knockdown abrogated NMN-mediated suppression of hyperpermeability and VE-cadherin degradation. Therefore, the protective effect of NMN against IL-1β-mediated endothelial dysfunction is dependent on the NAD^+^**-**SIRT1 axis.

**Conclusions:**

This *in vitro* mechanistic study suggests that the NAD⁺**-**SIRT1 axis contributes to IL-1β-induced endothelial barrier disruption, supporting further investigation of NMN in inflammatory vascular diseases.

## Introduction

Vascular endothelial dysfunction, particularly increased endothelial permeability, is a fundamental pathological process shared by a broad spectrum of inflammatory vascular disorders, including atherosclerosis, vasculitis, sepsis-associated vascular leakage, and diabetic microvascular complications (1–4). In healthy vasculature, the endothelial barrier tightly regulates the selective transmigration of solutes, macromolecules, and immune cells between circulating blood and adjacent tissues, thereby maintaining vascular homeostasis. Compromise of endothelial barrier integrity promotes excessive vascular leakage, inflammatory cell infiltration, and tissue injury, thereby contributing to disease initiation and progression across multiple vascular beds (5, 6).

Among these inflammatory vascular disorders, atherosclerosis represents a prototypical example in which endothelial hyperpermeability plays a critical initiating role (7). Increased endothelial permeability facilitates the infiltration of low-density lipoproteins (LDL) and other circulating inflammatory mediators into the subendothelial space, triggering lipid oxidation, monocyte recruitment, and macrophage activation. These macrophages engulf oxidized LDL to form foam cells, a hallmark of atherosclerotic plaques (8). Macrophages polarize into pro-inflammatory M1 or anti-inflammatory M2 phenotypes. M1 macrophages secrete pro-inflammatory cytokines, such as interleukin (IL)-1β, IL-6, and tumor necrosis factor-α (TNF-α), further propagating vascular inflammation (9). These inflammatory cytokines contribute to endothelial dysfunction by promoting oxidative stress, impairing nitric oxide production, and disrupting endothelial junction integrity, leading to increased vascular permeability and plaque instability (10). Notably, similar macrophage-endothelial interactions and cytokine-mediated barrier disruption occur in other inflammatory vascular diseases, indicating that mechanistic insights gained from atherosclerosis models may have broader translational relevance.

Vascular endothelial (VE)-cadherin is a key adherens junction protein crucial for maintaining endothelial cell-cell adhesion and vascular integrity (11). It regulates endothelial barrier permeability, preventing excessive infiltration of lipoproteins and immune cells into the vessel wall. In atherosclerosis, multiple pathological stimuli, including inflammatory cytokines, oxidative stress, and disturbed blood flow, promote VE-cadherin internalization and degradation, weaken intercellular junctions, and enhance endothelial permeability (12).

Nicotinamide adenine dinucleotide (NAD^+^) is an essential coenzyme involved in cellular metabolic processes and a crucial cosubstrate for various enzymes. NAD^+^ depletion is linked to vascular dysfunction and observed in several cardiovascular diseases (CVDs), including heart failure, ischaemia–reperfusion injury, arrhythmia, and atherosclerosis (13, 14). Additionally, NAD^+^-dependent enzymes, such as sirtuin (SIRT), help preserve endothelial integrity by regulating gene expression and cellular stress responses (15, 16). Accordingly, restoration of NAD^+^ levels through supplementation with NAD^+^ precursors has attracted attention as a potential approach to ameliorate endothelial dysfunction across a spectrum of vascular inflammatory diseases (17).

Nicotinamide mononucleotide (NMN), a key NAD^+^ biosynthesis intermediate, effectively elevates NAD^+^ levels *in vitro*, *in vivo*, and in clinical studies (18–20). Our previous randomized clinical trial showed that pulse wave velocity (PWV) values indicating arterial stiffness tended to decrease in the NMN intake group, yet not significantly, compared with those in the placebo group, suggesting a potential beneficial effect on vascular function (21). Animal studies further support the vascular protective and anti-inflammatory effects of NMN. In aged mice, oral NMN supplementation restores arterial SIRT1 activity and ameliorates age-related arterial dysfunction (22). In high-fat diet-fed ApoE^−/−^ mice, NMN attenuates vascular inflammation and reduces macrophage-associated pro-inflammatory cytokine expression within the arterial wall (23). Additionally, NMN suppresses lipopolysaccharide (LPS)-induced macrophage inflammation by inhibiting the cyclooxygenase 2 (COX2)-prostaglandin E2 pathway (24). These studies indicate that NMN modulates macrophage-driven inflammation and vascular dysfunction. However, the mechanisms through which NMN protects endothelial barrier integrity against macrophage-derived inflammatory stimuli remain incompletely understood.

In this study, we constructed a three-dimensional (3D) co-culture model of human umbilical vein endothelial cells (HUVECs) and M1 macrophages to verify whether NMN protects VE cells from macrophage-derived inflammatory stimuli. By focusing on IL-1β–induced endothelial hyperpermeability and VE-cadherin degradation, we explored the molecular mechanisms underlying the protective effects of NMN on endothelial barrier function.

## Materials and methods

### Cell cultures

HUVECs obtained from Lonza (Basel, Switzerland) were cultured in KBM VEC-1 basal medium (Kohjin Bio, Saitama, Japan) containing KBM VEC-1 supplement (Kohjin Bio) and 2% fetal bovine serum (FBS) at 37°C in a humidified atmosphere with 5% CO_2_. The cells were passaged when they reached 80%–90% confluence. THP-1 cells (Japanese Collection of Research Bioresources Cell Bank, Osaka, Japan) were cultured in RPMI 1640 medium (Nacalai Tesque Inc., Kyoto, Japan) with 10% FBS at 37°C under a humidified atmosphere of 5% CO_2_. THP-1 cells were plated in 24-well plates and differentiated into M0 macrophages with 100 nM phorbol 12-myristate 13-acetate (Merck/Sigma-Aldrich, Darmstadt, Germany) for 72 h. Cells were subsequently treated with 10 µg/mL LPS (Merck/Sigma-Aldrich) for 30 min to induce M1 macrophage differentiation.

### Preparation of a 3D co-culture model

HUVECs were seeded on cell culture inserts (pore size 1 μm, Corning Falcon, NY). After 48 h of incubation, inserts were transferred to 24-well plates seeded with LPS-differentiated M1 macrophages. After 48 h of co-culture in a medium with 1:1 KBM VEC-1 and RPMI 1640, HUVEC monolayers on inserts were transferred to 24-well plates without macrophages for endothelial permeability testing.

### Permeability test

The medium in the upper chamber was replaced with 200 μL medium containing 2 μg FITC-labelled 70 kDa dextran and Dil-labelled 500 kDa LDL (both from Thermo Fisher Scientific, Waltham, MA). The bottom chamber was filled with 400 μL fresh medium. After 60 min, 200 μL of medium from the bottom chamber was sampled. The fluorescence of FITC-dextran and Dil-LDL was measured using an Infinite 200 Pro plate reader (Tecan, Männedorf, Switzerland), and the excitation/emission wavelengths were set at 490/520 and 547/576 nm, respectively. FITC and Dil fluorescence passing through cell-free culture inserts was set as 100% transmittance, and sample permeability was calculated accordingly.

### Quantitative polymerase chain reaction (PCR)

Total RNA was extracted from cultured cells using the RNeasy Mini Kit (Qiagen, Mississauga, Canada) and stored at −80°C. To create first-strand cDNA, 1 μg of total RNA was used with the PrimeScript II 1st strand cDNA Synthesis Kit (Takara Bio, Shiga, Japan). Target mRNA expression levels were measured using an Applied Biosystems 7,500 Real-Time PCR System (Applied Biosystems, Foster City, CA) and *CD86* (assay ID: Hs.PT.58.21526437), *CD80* (assay ID: Hs.PT.56a.38577902), *CD206* (assay ID: Hs.PT.58.15093573), *CD163* (assay ID: Hs.PT.58.3564170), *VCAM-1* (assay ID: Hs.PT.58.20405152), *COX2* (assay ID: Hs.PT.58.77266), *IL-6* (assay ID: Hs.PT.58.40226675), *GAPDH* (assay ID: Hs.PT.39a.22214836), and *IL-1*β (assay ID: Hs.PT.58.1518186) PrimeTime® Std qPCR Assays. *GAPDH* served as an internal control. Relative quantification of target gene expression was performed using the *ΔΔ*Ct method.

### Enzyme-linked immunosorbent assay (ELISA)

Macrophages seeded in 24-well culture plates were activated with LPS for 30 min. The culture medium was subsequently replaced with fresh medium and the cells were incubated for 2–48 h. Human IL-1β and TNF-α in the M1 macrophage culture medium were quantified using human IL-1β and TNF-α Quantikine ELISA kits, respectively (R&D Systems, Minneapolis, MN) according to the manufacturer's instructions.

### Fluorescence microscope analysis

HUVECs were collected and washed thrice with phosphate-buffered saline (PBS), followed by fixation in 4% paraformaldehyde in PBS for 20 min at room temperature (RT). After three additional PBS washes, cells were permeabilized with 0.1% Triton X-100 in PBS for 10 min at RT. Cells were subsequently washed three times with PBS, blocked with 5% skim milk in Tris-buffered saline with 0.1% Tween-20 (TBS-T) for 30 min at RT, and washed three times with TBS-T. Cells were subsequently stained for VE-cadherin and NF-*κ*B/p65 with 1:350 diluted anti-VE-cadherin (#sc-9989, Santa Cruz Biotechnology, CA) and anti-NF-*κ*B p65 antibodies (#8242, Cell Signaling Technology, Danvers, MA), respectively, overnight at 4°C. Thereafter, the cells were washed thrice with TBS-T and incubated with 1:400 diluted Alexa Fluor 488 anti-mouse IgG or Alexa Fluor 594 anti-rabbit IgG antibodies for 1 h at RT. For nuclear and F-actin staining, cells were treated with a 1:400 dilution of 4ʹ-6-diamidino-2-phenylindole (DAPI) solution and Alexa Fluor 594 phalloidin for 20 min at RT. After three washes with TBS-T, fluorescence images were recorded using a fluorescence microscope BZ-X800 (Keyence, Osaka, Japan). The area of the intercellular gap was measured after binarizing nine randomly selected F-actin-stained images with a global threshold using ImageJ software. The percentage of NF-*κ*B/p65 co-localisation with the nuclei was quantified using BZ-H4C/hybrid cell count software. For statistical analysis, >1,000 HUVECs were observed in randomly selected images.

### Immunoblotting

HUVECs were lysed with RIPA lysis buffer (150 mM sodium chloride, 1% NP-40, 0.5% sodium deoxycholate, 0.1% SDS, 50 mM Tris, pH 8.0) containing protease (Hoffmann-La Roche, Basel, Switzerland) and phosphatase inhibitor cocktails (Thermo Fisher Scientific). Equal amounts of protein were electrically separated using an 8% SDS-polyacrylamide gel and subsequently transferred to PVDF membranes. Membranes were blocked with 5% skim milk in TBS-T for 1 h at RT. Thereafter, the membranes were incubated with monoclonal antibodies against VE-cadherin (#sc-9989, Santa Cruz Biotechnology) and *β*-actin (Abcam, Cambridge, MA) overnight at 4°C. After three TBS-T washes, membranes were incubated with horseradish peroxidase-conjugated goat anti-mouse and goat antibodies for 3 h. Immunoreactive bands were detected using the ECL Prime Western blotting Detection Reagent (Cytiva, Tokyo, Japan) and captured with an ImageQuant LAS 4000 (GE Healthcare, Tokyo, Japan).

### NAD^+^ quantification

The NAD^+^ assay was performed using the NAD^+^/NADH Assay Kit-WST (Dojindo Laboratories). HUVECs were lysed with NAD^+^/NADH Extraction Buffer and filtered using a kit-provided ultracentrifugal filter for slow proteins. The filtrate was split into two portions and either kept on ice to measure total NAD+/NADH or subjected to heat treatment at 60°C for 60 min to measure NADH. Both samples were incubated with the working solution at 37°C for 60 min. Absorbance at 450 nm was measured using an Infinite 200 Pro plate reader (Tecan). NAD^+^ levels were calculated by subtracting the NADH levels from the total NAD^+^/NADH levels and were normalised to their respective protein concentrations determined using the Pierce BCA protein assay (Thermo Fisher Scientific).

### Small interfering RNA (siRNA) transfection

HUVECs were transfected with the ON-TARGETplus Non-targeting Pool (No. D-001810-10-20, Dharmacon, Lafayette, CO) or ON-TARGETplus SIRT1 siRNA (No. L-003540-00-0005, Dharmacon) using HiPerFect Transfection Reagent (Qiagen). Based on the results of preliminary tests evaluating knockdown efficiency (Supplementary Figure S4), the concentrations of siRNA and transfection reagent were set to 300 ng/mL and 3 μL/mL, respectively.

### Statistical analyses

Data are presented as mean ± standard error of the mean (SEM), derived from a minimum of three independent experiments. Differences among multiple groups were determined using one-way analysis of variance (ANOVA), followed by the Tukey–Kramer test using Statcel software (third edition; OMS, Tokyo, Japan). Statistical significance was set at *p* < 0.05. Asterisks and daggers denote statistical significance compared with the control (*p < 0.05, **p < 0.01) and the indicated groups (^†^*p* < 0.05, ^††^*p* < 0.01).

## Results

### NMN attenuated HUVEC hyperpermeability induced by co-culture with M1 macrophages

**Figure 1:**
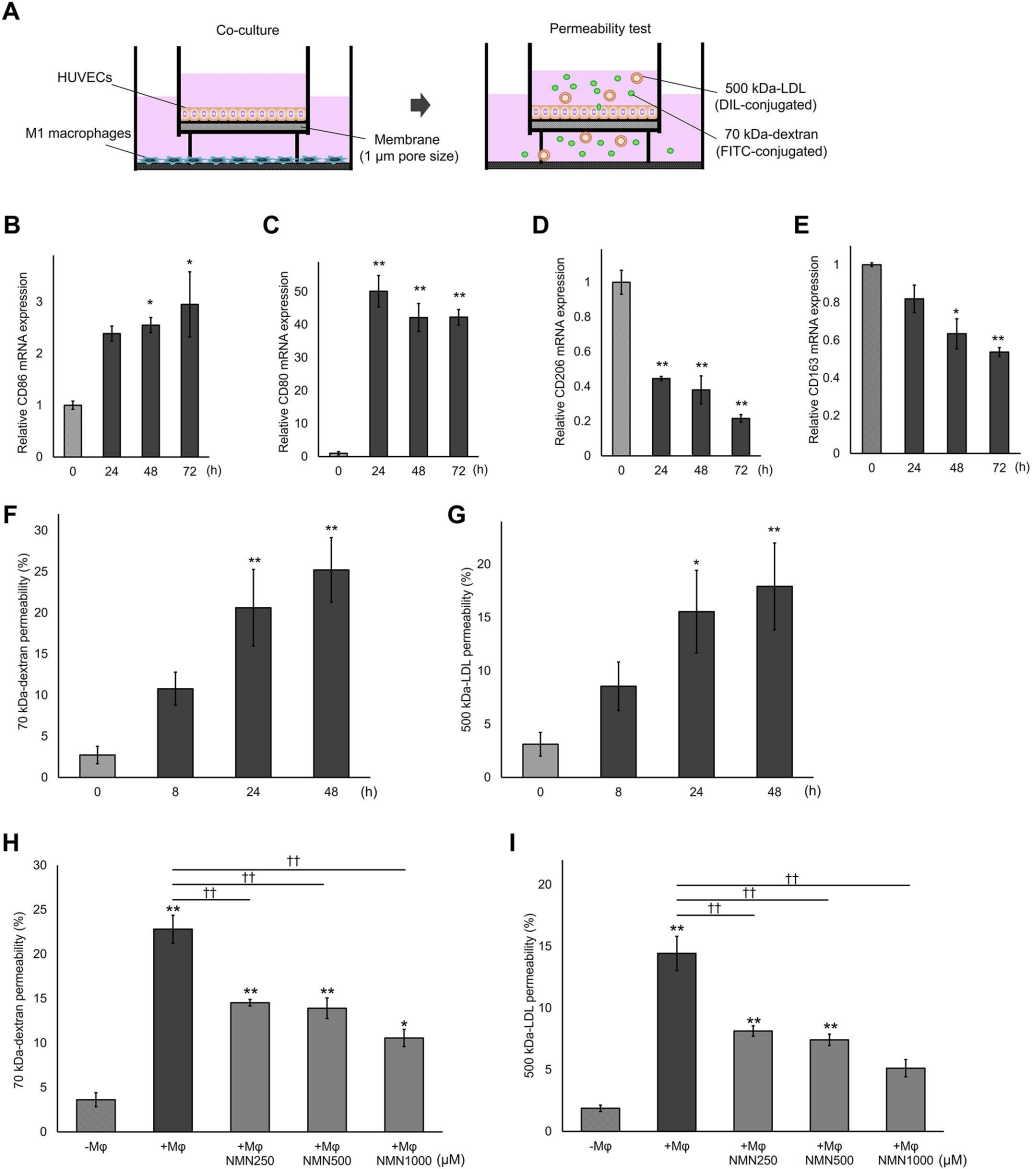
NMN ameliorates endothelial hyperpermeability induced by M1 macrophage co-culture. **(A)** HUVECs were plated onto cell culture inserts consisting of 1 µm pore membranes and co-cultured with M1-macrophages seeded on the underside of 24-well plates for 0–48 h. **(B–E)** Macrophages were stimulated with 10 µg/mL LPS for 30 min and incubated for 0–72 h. Thereafter, their *CD86*, *CD80*, *CD206*, and *CD163* mRNA expression levels were determined using qPCR. **(F,G)** Endothelial permeability tests were performed by quantifying 70 kDa-dextran and 500 kDa-LDL levels in the lower chamber. **(H,I)** HUVECs plated onto cell culture inserts were pretreated with NMN (250–1,000 µM) for 24 h and co-cultured with M1-macrophages (M*Φ*) for 48 h. Thereafter, 70 kDa-dextran and 500 kDa-LDL permeability were quantitated. *p < 0.05 and **p < 0.01, compared with zero time or macrophage absence (-M*Φ*). ^††^*p* < 0.01, compared with macrophage presence (+M*Φ*). Data are presented as mean ± SEM; *n* = 3 independent experiments, each performed in triplicate **(B–E,G–I)**; *n* = 4 independent experiments, each performed in triplicate **(F)**.

M1 macrophages secrete pro-inflammatory cytokines that considerably alter VE permeability (10). To assess how the cytokine environment produced during LPS-mediated M0-to-M1 macrophage polarization affects endothelial monolayer integrity and permeability, a 3D co-culture model of HUVECs and M1 macrophages was constructed (Figure 1A). M1 macrophages were polarized through LPS stimulation, and M1/M2 polarization was confirmed by quantifying the gene expression levels of each marker. The expression of M1 markers *CD86* and *CD80* in macrophages gradually increased after LPS stimulation, and both reached significantly high levels after 48 h (Figures 1B,C). Conversely, *CD206* and *CD163* levels significantly decreased 48 h after stimulation (Figures 1D,E). Endothelial permeability was evaluated by measuring the passage of 70 kDa dextran and 500 kDa LDL from the luminal (top) to the abluminal side (bottom) of the insert (Figure 1A). Co-culture with M1 macrophages increased HUVEC permeability in a time-dependent manner, with significant differences observed at 24 and 48 h (Figures 1F,G). These results showed that cytokines released during LPS-induced M0-to-M1 macrophage polarization increased endothelial monolayer permeability. Our previous studies have shown that NMN supplementation suppresses hydrogen peroxide-induced cytoskeletal disruption by preventing NAD^+^ depletion in HUVECs (25, 26), suggesting that NMN protects endothelial hyperpermeability under oxidative stress. To determine whether NMN protects endothelial cells from inflammation-induced hyperpermeability, the effect of NMN pretreatment on HUVECs co-cultured with M1 macrophages was evaluated. Notably, 24 h NMN pretreatment prevented M1 macrophage-induced 70 kDa-dextran hyperpermeability in HUVECs (Figure 1H). Similarly, NMN pretreatment reduced 500 kDa-LDL hyperpermeability induced by M1 macrophages (Figure 1I). These results suggest that NMN protects endothelial cells from hyperpermeability induced by pro-inflammatory cytokines released during M1 macrophage polarization.

### NMN reduced the hyperpermeability induced by M1 macrophage-released IL-1β

**Figure 2:**
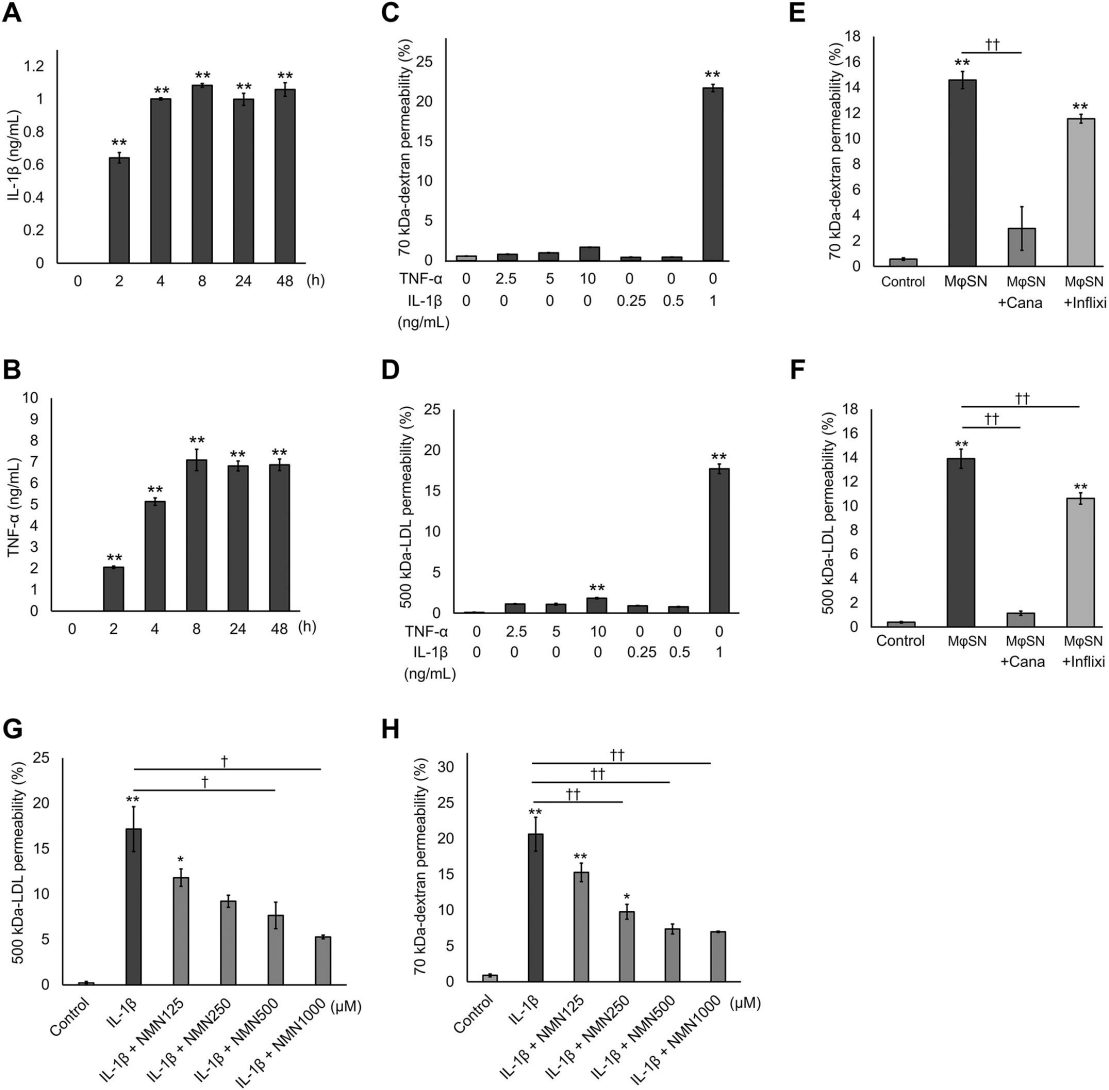
NMN reduces hyperpermeability induced by M1 macrophage-derived IL-1β. **(A,B)** Macrophages seeded in 12-well plates were stimulated with 10 µg/mL LPS for 30 min and incubated for 0–72 h. Thereafter, IL-1β and TNF-α levels in the supernatant were measured using ELISA. **(C,D)** HUVECs plated on cell culture inserts were incubated with IL-1β (0.25–1 ng/mL) or TNF-α (2.5–10 ng/mL) for 48 h. Next, 70 kDa-dextran and 500 kDa-LDL permeability were quantitated. **(E,F)** HUVECs plated on cell culture inserts were incubated with the M1 macrophage culture supernatant (M*Φ*SN) in the presence or absence of 100 µg/mL IL-1β neutralizing antibody canakinumab (Cana) or TNF-α neutralizing antibody infliximab (Inflixi) for 48 h. Thereafter, 70 kDa-dextran and 500 kDa-LDL permeability were quantitated. **(G,H)** HUVECs plated on cell culture inserts were pretreated with NMN (125–1,000 µM) for 24 h and incubated with 1 ng/mL IL-1β for 48 h. Thereafter, 70 kDa-dextran and 500 kDa-LDL permeability were quantitated. *p < 0.05 and **p < 0.01, in comparison with zero time or control. ^†^*p* < 0.05 and ^††^*p* < 0.01, compared with the indicated groups. Data are presented as mean ± SEM; *n* = 3 independent experiments, each performed in triplicate **(A–D,F–H)**; *n* = 4 independent experiments, each performed in triplicate **(E)**.

IL-1β and TNF-α can disrupt junctional molecules and increase endothelial permeability (27). Following LPS stimulation, IL-1β (0.6–1.1 ng/mL) and TNF-α (2–7 ng/mL) were detected in the culture supernatant of M1-polarizing macrophages over the 2–48 h observation period (Figures 2A,B). Experiments using recombinant and neutralizing antibodies against each cytokine were performed to determine which M1 macrophage-released cytokine most affected endothelial permeability. Recombinant IL-1β (1 ng/mL) significantly increased 70 kDa-dextran permeability, whereas TNF-α (2.5–10 ng/mL) had no effect (Figure 2C). Although both 1 ng/mL IL-1β and 10 ng/mL TNF-α significantly enhanced 500 kDa-LDL permeability, the effect of TNF-α was limited compared with that of IL-1β (Figure 2D). Additionally, the IL-1β neutralizing antibody canakinumab successfully reduced 70 kDa-dextran hyperpermeability induced by the M1 macrophage supernatant (Figure 2E). In contrast, the TNF-α neutralizing antibody infliximab reduced hyperpermeability; however, this was not significant (Figure 2E). The IL-1β antibody completely blocked 500 kDa-LDL hyperpermeability induced by the M1 macrophage supernatant, whereas the TNF-α antibody caused only moderate inhibition (Figure 2F). These results showed that under our experimental conditions, M1 macrophage-released IL-1β primarily contributes to endothelial hyperpermeability. We also confirmed that NMN suppressed recombinant IL-1β-induced endothelial hyperpermeability in a dose-dependent manner (Figures 2G,H). Therefore, these results indicate that NMN protects HUVECs from hyperpermeability induced by M1 macrophage-derived IL-1β.

### NMN suppressed gap formation and VE-cadherin degradation induced by M1 macrophage-released IL-1β

**Figure 3:**
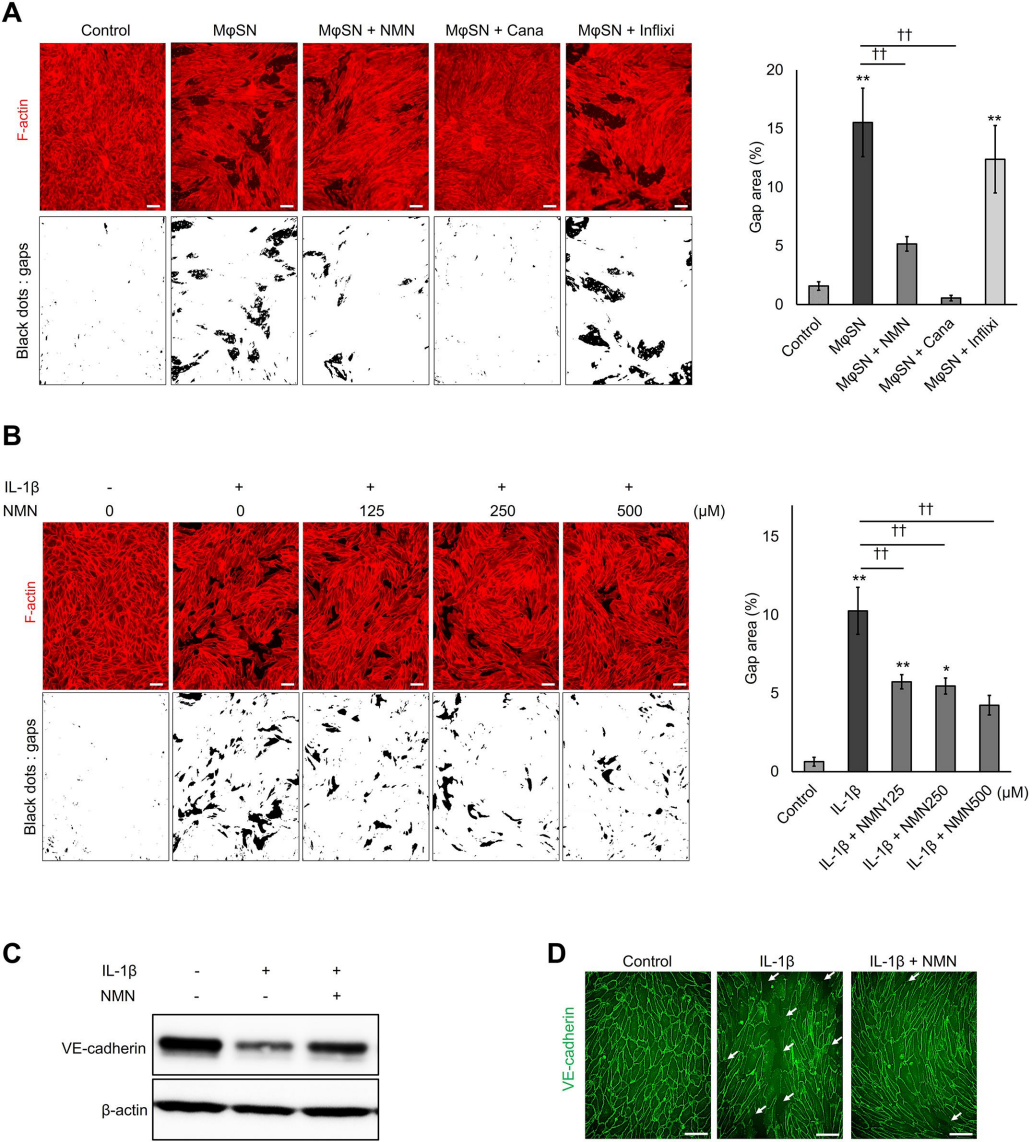
NMN inhibits IL-1β-induced adherens junction disassembly. **(A)** HUVECs plated on cell culture inserts were pretreated with 500 µM NMN for 24 h and incubated with the M1 macrophage culture supernatant (M*Φ*SN) in the presence or absence of 100 µg/mL IL-1β neutralizing antibody canakinumab (Cana) or TNF-α neutralizing antibody infliximab (Inflixi) for 48 h. The F-actin cytoskeleton was stained with Alexa Fluor 594 phalloidin (red). Captured images were binarized using a global threshold. Black dots in the adjusted images represent intercellular gaps in the HUVEC monolayer. Intracellular gap areas were measured as the ratio of the black dot area to the entire image using ImageJ software. **(B)** HUVECs plated on cell culture inserts were pretreated with NMN (125–500 µM) for 24 h and incubated with 1 ng/mL IL-1β for 48 h. The F-actin cytoskeleton was stained (red), and the intracellular gap areas were calculated. **(C,D)** HUVECs seeded in 12- or 48-well plates were pretreated with 500 µM NMN for 24 h and incubated with 1 ng/mL IL-1β for 48 h. **(C)** Intracellular VE-cadherin levels were evaluated using western blotting with an anti-VE-cadherin antibody. *β*-actin served as a loading control. **(D)** Cell-to-cell adherens junctions were visualized by staining with VE-cadherin (green). White arrows in the images represent adherens junction discontinuity. Scale bar: 100 μm. *p < 0.05 and **p < 0.01, compared with the control. ^††^*p* < 0.01, compared with the indicated groups. Representative blot and immunofluorescence images are shown **(A–D)**. Data are presented as mean ± SEM; *n* = 3 independent experiments, each based on the analysis of ≥5 images per condition **(A,B)**; *n* = 5 independent experiments **(C)**; *n* = 3 independent experiments **(D)**.

One of the mechanisms by which IL-1β induces vascular hyperpermeability is by altering the expression of cell-cell junction components, resulting in the formation of small holes and gaps between cells (28). To confirm whether NMN suppresses macrophage-released IL-1β-induced intercellular gaps in HUVECs, the F-actin cytoskeleton was stained with fluorescently labelled phalloidin, and the gap area between cells was calculated. In subsequent tests, the maximum NMN concentration was set at 500 µM, showing a significant inhibitory effect in all previous tests (Figures 1H, 2G,H). The M1 macrophage supernatant generated an intercellular gap area of approximately 15%, whereas NMN or IL-1β neutralizing antibody canakinumab pretreatment significantly inhibited gap area expansion (Figure 3A). Moreover, NMN significantly reduced recombinant IL-1β-induced gap formation in a concentration-dependent manner (Figure 3B). These results suggest that NMN protects against endothelial cell hyperpermeability by preventing intercellular gap formation induced by M1 macrophage-released IL-1β.

VE-cadherin is an endothelial cell-specific component of adherens junctions that plays an essential role in maintaining vascular integrity (11). Therefore, VE-cadherin disorganization increases endothelial permeability (29). IL-1β markedly decreases the expression and staining of VE-cadherin in HUVECs (30, 31). To examine whether NMN affects adherens junction assembly, VE-cadherin expression and cellular distribution were compared in IL-1β-stimulated HUVECs, with and without NMN pretreatment. Western blot analysis revealed that IL-1β stimulation reduced VE-cadherin protein levels, which were protected by NMN pretreatment (Figure 3C). VE-cadherin gene expression levels were also measured using qPCR, and no changes were observed in VE-cadherin mRNA after IL-1β stimulation (Supplementary Figure S1). These results suggest that NMN inhibits IL-1β-induced VE-cadherin degradation and does not affect VE-cadherin expression. Changes in intracellular VE-cadherin state were confirmed using immunofluorescence staining. In confluent monolayers of unstimulated HUVECs, VE-cadherin staining showed close localization between neighboring cells, indicating a narrow adherens junction architecture (Figure 3D left panel). In contrast, continuous VE-cadherin staining was disrupted and small gaps at cell-cell junctions were observed after IL-1β stimulation (white arrows). This interrupted VE-cadherin staining in IL-1β-stimulated cells was ameliorated by NMN pretreatment, confirming its protective effect against VE-cadherin disassembly (Figure 3D right panel). These findings support that NMN prevents adherens junction disorganization components induced by M1 macrophage-released IL-1β, thereby protecting against endothelial hyperpermeability from intercellular gap formation.

### NMN reduced IL-1β-induced hyperpermeability by inhibiting NF-*κ*B activation

**Figure 4:**
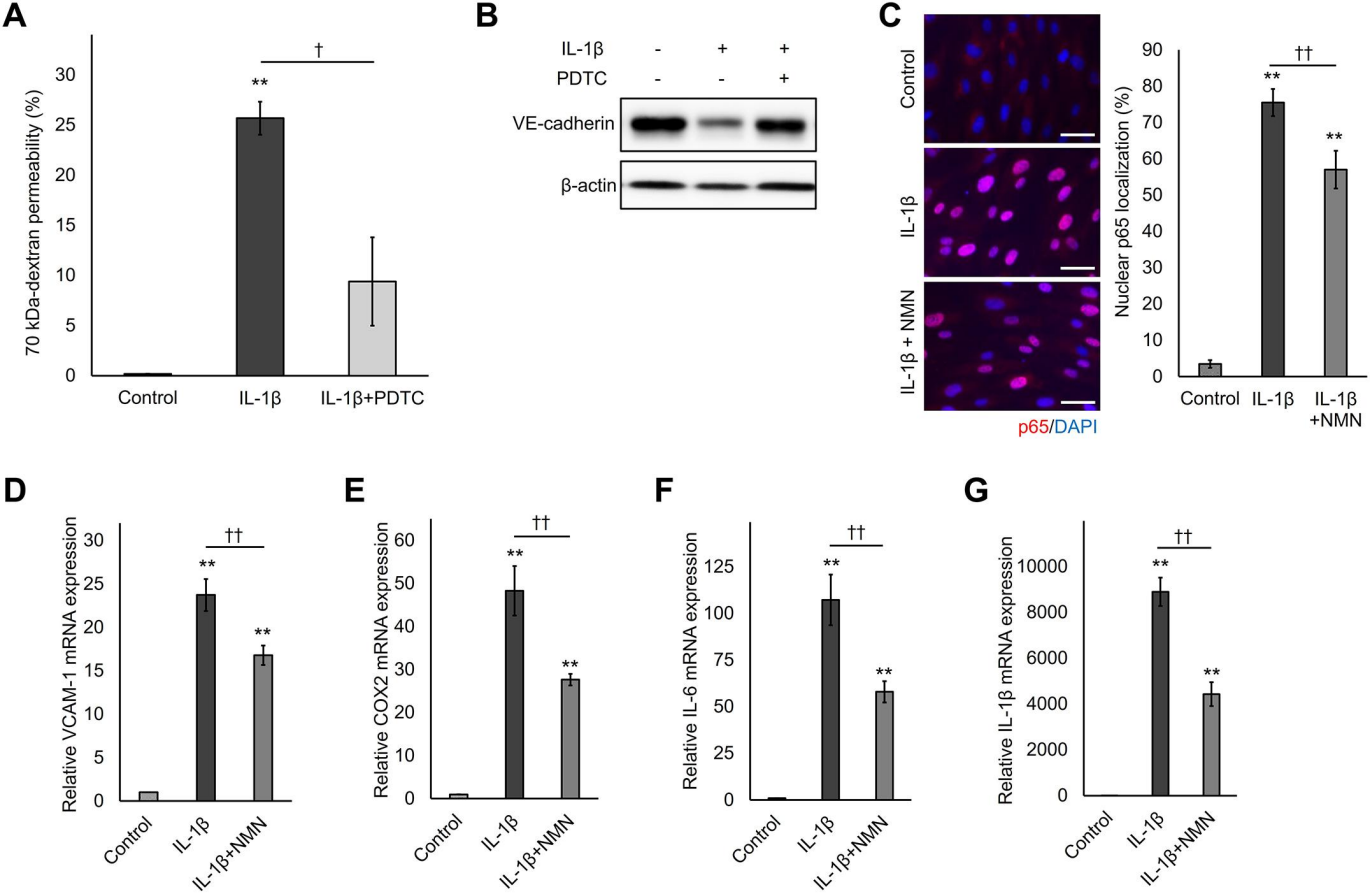
NMN diminishes IL-1β-induced endothelial hyperpermeability by suppressing NF-*κ*B activation. **(A)** HUVECs plated on cell culture inserts or 12 well-plates were pretreated with 100 µM NF-*κ*B inhibitor (PDTC) for 30 min. Thereafter, 1 ng/mL IL-1β was added and the cells were further cultured for 48 h. The 70 kDa-dextran permeability was determined using an endothelial permeability test. **(B)** Intracellular VE-cadherin levels were evaluated using western blotting with an anti-VE-cadherin antibody. *β*-actin was used as a loading control. **(C)** HUVECs seeded in 48-well plates were pretreated with 500 µM NMN for 24 h and incubated with 1 ng/mL IL-1β for 4 h. NF-*κ*B/p65 localization was visualized by staining with p65 (red), and the nuclei were counterstained with DAPI (blue). The co-localization of NF-*κ*B/p65 with the nuclei was quantified using BZ-H4C/hybrid cell count software. Scale bar: 50 μm. **(D–G)** HUVECs seeded in 12-well plates were pretreated with 500 µM NMN for 24 h and incubated with 1 ng/mL IL-1β for 24 h. mRNA expression levels of *VCAM-1*, *COX2*, *IL-6*, and *IL-1*β were determined using qPCR. **p < 0.01, compared with the control. ^†^*p* < 0.05 and ^††^*p* < 0.01, compared with the indicated groups. Representative blot and immunofluorescence images are shown **(B,C)**. Data are presented as mean ± SEM; *n* = 3 independent experiments, each performed in triplicate **(A)**; *n* = 3 independent experiments, each based on the analysis of ≥5 images per condition **(C)**; *n* = 4 independent experiments, each performed in triplicate **(D,G)**; *n* = 7 independent experiments, each performed in triplicate **(E,F)**; *n* = 3 independent experiments **(B)**.

The NF-*κ*B pathway is activated by various pro-inflammatory stimuli, including IL-1β. Furthermore, several NF-*κ*B pathway downstream factors affect VE-cadherin state (11), although no reports directly examining NF-*κ*B pathway involvement in IL-1β-induced VE-cadherin degradation exists. Therefore, to determine whether NF-*κ*B is involved in IL-1β-induced hyperpermeability, the NF-*κ*B inhibitor pyrrolidine dithiocarbamate (PDTC) was used. PDTC pretreatment noticeably suppressed IL-1β-induced 70 kDa-dextran hyperpermeability (Figure 4A). Additionally, decreased VE-cadherin protein by IL-1β stimulation was ameliorated by the NF-*κ*B inhibitor (Figure 4B). PDTC also decreased IL-1β-induced cell-cell gap formation (Supplementary Figure S2). These results suggest that IL-1β enhances endothelial permeability by activating the NF-*κ*B pathway.

NMN regulates NF-*κ*B activation by increasing SIRT1 activity (32). Therefore, we hypothesized that NMN protects HUVECs from IL-1β-induced hyperpermeability by inhibiting NF-*κ*B activation. To test this hypothesis, NF-*κ*B activation was evaluated by detecting NF-*κ*B/p65 nuclear translocation and downstream gene expression in NMN- and IL-1β-treated HUVECs. After IL-1β stimulation, noticeable NF-*κ*B/p65 nuclear translocation was detected, whereas NMN pretreatment decreased nuclear NF-*κ*B/p65 localization (Figure 4C). Additionally, qPCR analysis showed that NMN significantly inhibited IL-1β-induced expression of NF-*κ*B target genes, such as *VCAM-1*, *COX2*, *IL-6*, and *IL-1*β (Figures 4D–G). These downstream genes reduce the expression of adherens junction components, such as VE-cadherin, and increase endothelial permeability (31, 33–36). Therefore, our results indicated that NMN suppresses NF-*κ*B pathway activation induced by IL-1β stimulation, resulting in endothelial hyperpermeability inhibition associated with adherens junction disruption.

### NMN inhibited IL-1β-induced VE-cadherin degradation and hyperpermeability through a NAD^+^-SIRT1 axis dependent mechanism

**Figure 5:**
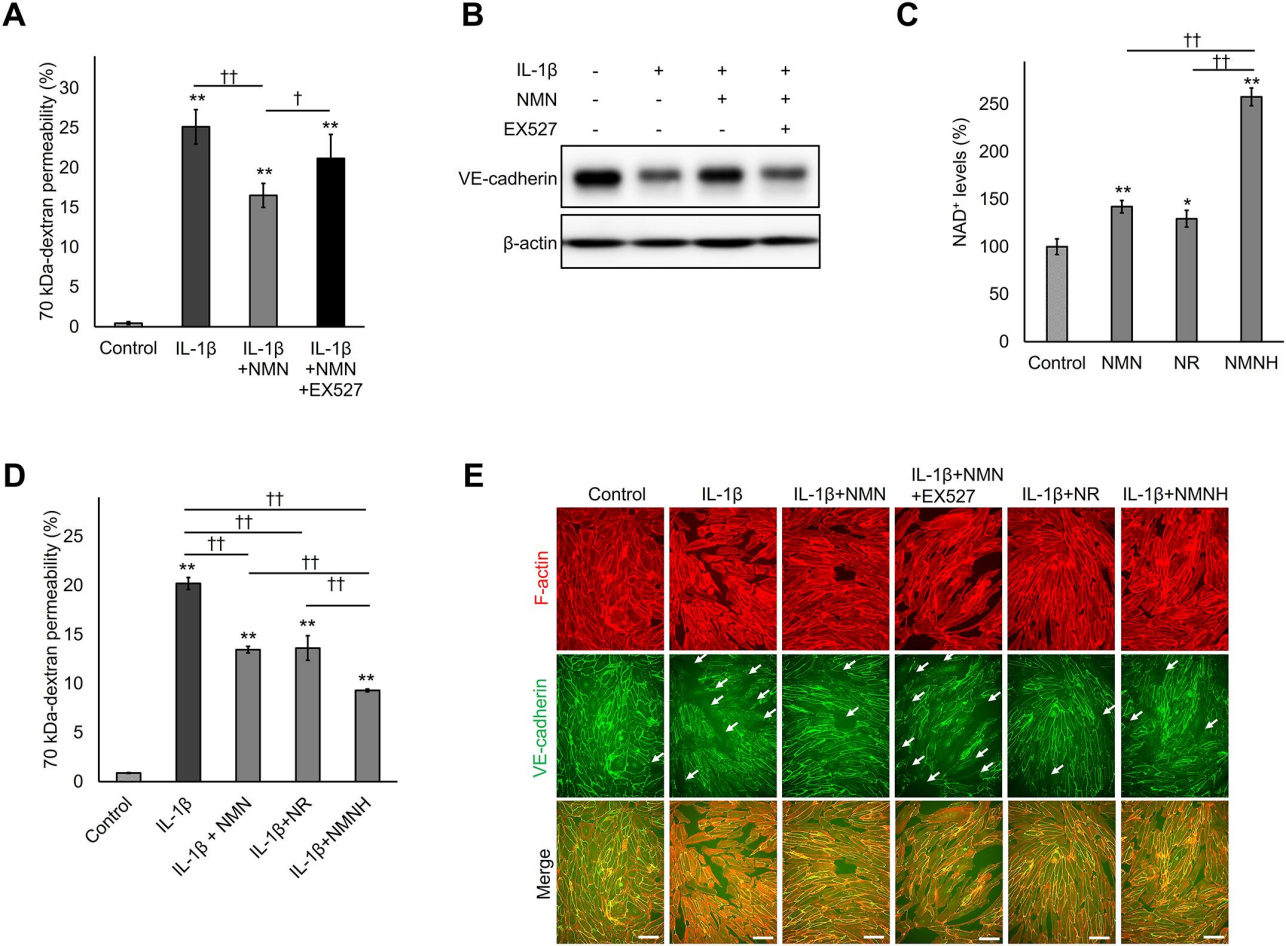
Effects of NMN on HUVECs were dependent on SIRT1 and NAD^+^ levels. HUVECs were seeded on cell culture inserts or 12-well plates, pretreated with 500 µM NMN for 24 h, and incubated with 1 ng/mL IL-1β for 48 h in the presence or absence of the 10 µM SIRT1 inhibitor EX527. **(A)** Next, 70 kDa-dextran permeability was determined using an endothelial permeability test. **(B)** Intracellular VE-cadherin levels were evaluated using western blotting with an anti-VE-cadherin antibody. *β*-actin served as a loading control. **(C)** HUVECs seeded in 24-well plates were incubated with 500 µM NMN, NR, or NMNH for 24 h, and intracellular NAD^+^ levels were determined using an NAD^+^/NADH assay kit. **(D)** HUVECs seeded on cell culture inserts were pretreated with 500 µM NMN, NR, or NMNH for 24 h and incubated with 1 ng/mL IL-1β for 48 h. Thereafter, 70 kDa-dextran permeability was determined using an endothelial permeability test. **(E)** HUVECs seeded in 48-well plates were pretreated with 500 µM NMN, NR, or NMNH for 24 h and incubated with 1 ng/mL IL-1β for 48 h in the presence or absence of 10 µM SIRT1 inhibitor EX527. The F-actin cytoskeleton and adherens junctions were stained with Alexa Fluor 594 phalloidin (red) and antibody-specific VE-cadherin (green), respectively. White arrows in the images represent adherens junction discontinuity. Scale bar: 100 μm. *p < 0.05 and **p < 0.01, compared with the control. ^†^*p* < 0.05 and ^††^*p* < 0.01, compared with the indicated groups. Representative blot and immunofluorescence images are shown **(B,E)**. Data are presented as mean ± SEM; *n* = 6 independent experiments, each performed in triplicate **(A)**; *n* = 3 independent experiments, each performed in triplicate **(C,D)**; *n* = 3 independent experiments **(E)**.

**Figure 6:**
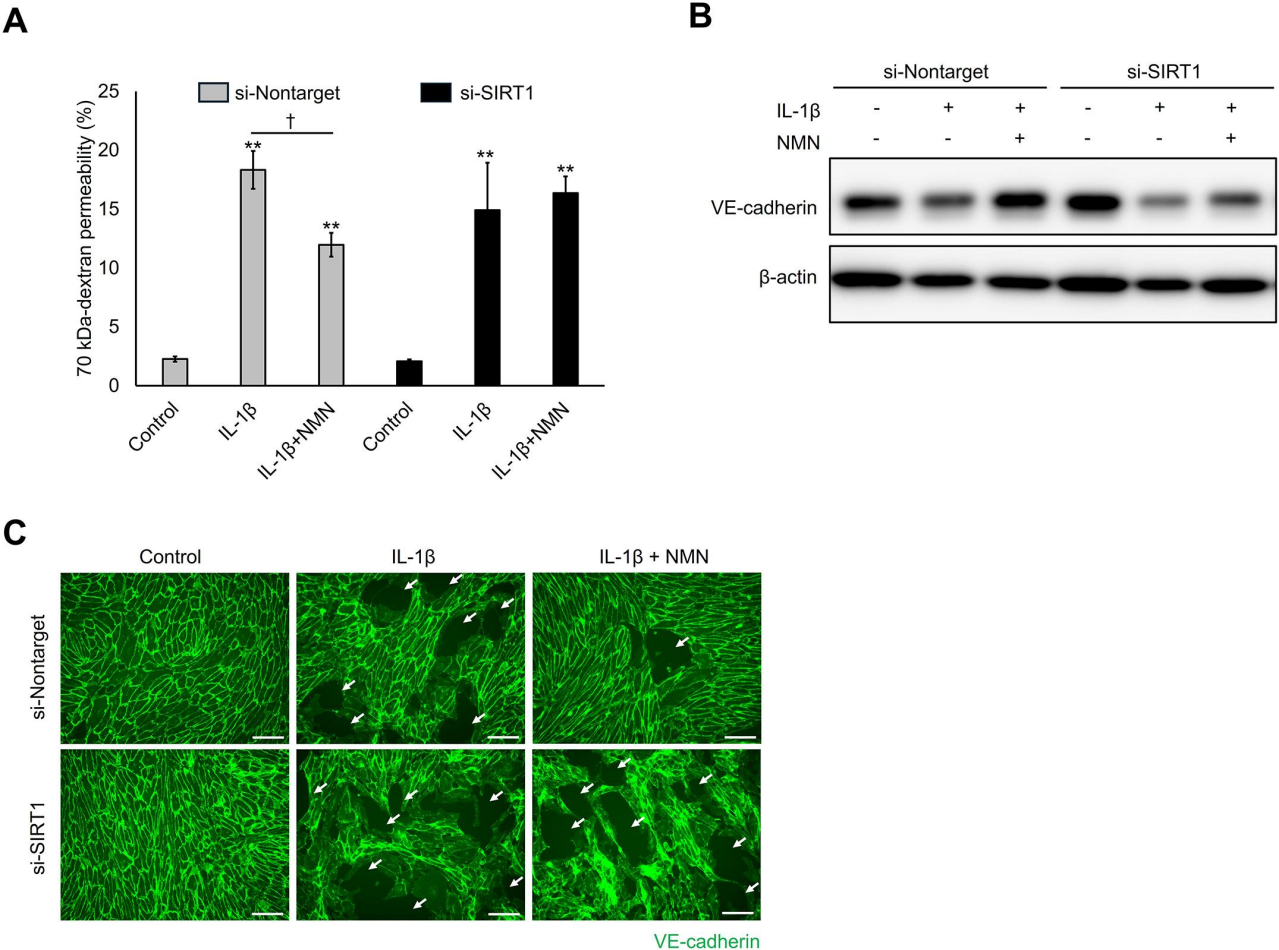
SIRT1-specific siRNA abolishes the permeability protection and VE-cadherin degradation inhibitory effect of NMN. HUVECs were transfected with the indicated siRNA (300 ng/mL) using HiPerFect Transfection Reagent for 24 h. The cells were pretreated with 500 µM NMN for 24 h and incubated with 1 ng/mL of IL-1β for 48 h. **(A)** Next,70 kDa-dextran permeability was determined using an endothelial permeability test. **(B)** Intracellular VE-cadherin levels were evaluated using western blotting with an anti-VE-cadherin antibody. *β*-actin served as a loading control. **(C)** Cell-to-cell adherens junctions were visualized by staining with VE-cadherin (green). White arrows in the images represent adherens junction discontinuity. Scale bar: 100 μm. **p < 0.01, compared with the control. ^†^*p* < 0.05, compared with the indicated groups. Representative blot and immunofluorescence images are shown **(B,C)**. Data are presented as mean ± SEM; *n* = 4 independent experiments, each performed in triplicate **(A)**; *n* = 3 independent experiments **(B,C)**.

NMN upregulates the enzymatic activity of SIRT1, an NAD^+^-dependent protein deacetylase, by increasing intracellular NAD^+^ levels. Additionally, SIRT1 inhibits the NF-*κ*B pathway through direct deacetylation of NF-*κ*B/p65, resulting in inflammatory response reduction (37). Therefore, to confirm whether the suppression of VE-cadherin degradation and subsequent hyperpermeability regulation of NMN were SIRT1-dependent, the effect of the SIRT1 inhibitor EX527 on the protective activity of NMN in IL-1β-stimulated HUVECs was examined. EX527 treatment significantly abrogated the protective effect-mediated suppression of NMN on IL-1β-induced hyperpermeability (Figure 5A). In addition, EX527 counteracted the restoration of VE-cadherin protein expression by NMN in IL-1β-stimulated cells (Figure 5B). These observations suggest that the regulatory effect of NMN on transendothelial permeability is SIRT1 dependent. Similar to NMN, reduced NMN (NMNH) and NR, which increased intracellular NAD^+^ levels (Figure 5C), also significantly inhibited IL-1β-induced endothelial hyperpermeability (Figure 5D). Notably, NMNH, which has stronger NAD^+^-enhancing activity than NMN (Figure 5C), also inhibited IL-1β-induced hyperpermeability more effectively than NMN (Figure 5D). These results indicate that increased intracellular NAD^+^ levels correlate with its ability to inhibit IL-1β-induced VE permeability enhancement. VE-cadherin and F-actin staining was used to evaluate the effects of SIRT1 inhibitor EX527 and the NAD^+^ precursors NR and NMNH on IL-1β-induced cell-cell gap formation and VE-cadherin localisation. EX527 treatment exacerbated gap formation and VE-cadherin discontinuity in NMN- and IL-1β-treated cells (Figure 5E). Similar to NMN, NR and NMNH inhibited IL-1β-induced gap formation and VE-cadherin degradation (Figure 5E). Finally, SIRT1 dependence was confirmed using gene-targeting knockdown experiments. siRNA-mediated SIRT1 knockdown significantly counteracted the regulatory effect of NMN on hyperpermeability and VE-cadherin degradation induced by IL-1β (Figures 6A,B). Additionally, SIRT1 knockdown abolished the protective effect of NMN on VE-cadherin continuity (Figure 6C). Collectively, these results indicate that increased intracellular NAD^+^ levels and SIRT1 activity are crucial for NMN to protect VE cells from inflammatory stimuli-induced hyperpermeability.

## Discussion

**Figure 7:**
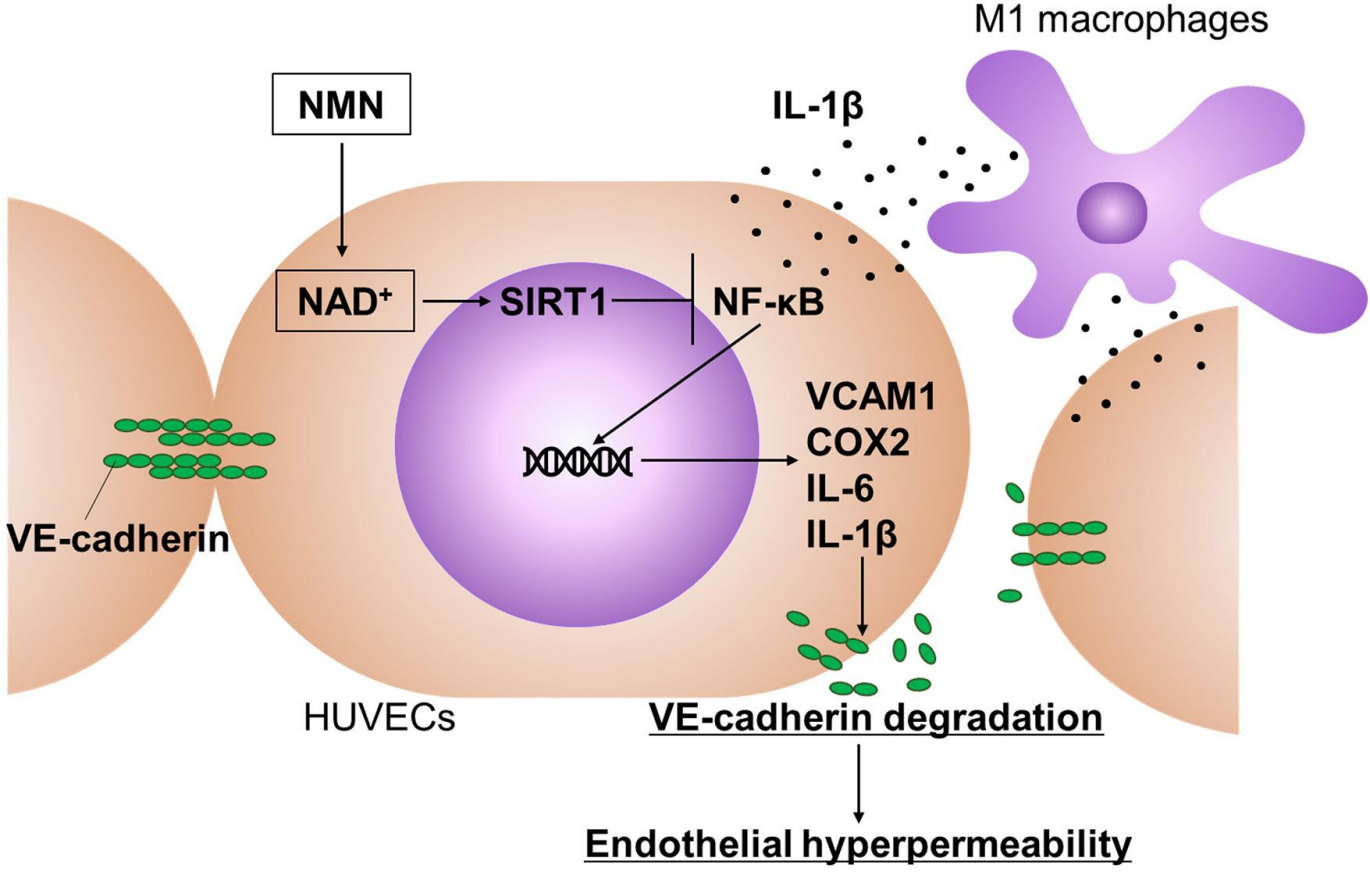
Molecular mechanism by which NMN protects vascular endothelial cells from endothelial hyperpermeability induced by M1 macrophage-derived IL-1β stimulation.

With an aging population, CVDs, such as atherosclerosis and other vascular inflammatory disorders, increasingly impact human health and life expectancy (38). Although NMN, an NAD^+^ precursor, has shown potential in improving vascular function, its underlying mechanisms remain unclear. In this study, we constructed a 3D co-culture model of VE cells and macrophages to reproduce inflammatory vascular microenvironments. After co-culture, endothelial permeability of 70 kDa-dextran and 500 kDa-LDL was evaluated. NMN suppressed endothelial hyperpermeability induced by M1 macrophage-derived cytokine inflammatory stress. We also identified a novel mechanism by which NMN inhibits VE-cadherin reduction in endothelial cells. The putative mechanism by which NMN maintains endothelial permeability at sites of vascular inflammation is described in Figure 7. At these sites, macrophages polarised into M1 macrophages, releasing various pro-inflammatory cytokines, including IL-1β. IL-1β elicits an inflammatory response in nearby endothelial cells and activates the NF-*κ*B pathway. Activated NF-*κ*B/p65 translocates into the nucleus, and its transcriptional activity increases target gene (*VCAM-1*, *COX2*, *IL-6*, and *IL-1*β) expression. These factors degrade adherens junction components, such as VE-cadherin, a key regulator of endothelial barrier integrity. VE-cadherin disassembly causes cell-cell adhesion instability, increasing endothelial permeability. NMN increases NAD^+^ levels, enhancing SIRT1 activity, which suppresses the NF-*κ*B pathway. Consequently, VE-cadherin homeostasis is maintained, protecting endothelial cells from increased permeability (Figure 7).

VE-cadherin is an endothelial-specific protein crucial for maintaining vascular endothelium barrier function. Barrier function disruption exacerbates vascular inflammatory diseases by promoting leukocyte extravasation into the vessel wall through paracellular diapedesis from the vascular lumen (39, 40). Thus, our finding that NMN suppresses VE-cadherin degradation and maintains adherens junction continuity in IL-1β-stimulated HUVECs (Figures 3C,D) provides mechanistic support for further investigation of NMN in endothelial barrier regulation under inflammatory conditions. VE-cadherin degradation is also associated with systemic inflammatory disease pathogenesis, such as sepsis and acute kidney injury (29, 41). Furthermore, human studies have demonstrated increased serum levels of soluble VE-cadherin, cleaved from the cell surface, in conditions associated with endothelial dysfunction, including systemic vasculitis, malignancy, and rheumatoid arthritis (42–44). VE cadherin degradation products are being explored as promising biomarkers of endothelial dysfunction in various diseases (45, 46). Therefore, our findings suggest that preservation of VE-cadherin may be relevant to endothelial barrier regulation under inflammatory conditions.

In this study, NMN suppressed VE-cadherin degradation by inhibiting the NF-*κ*B pathway. NF-*κ*B/p65 undergoes multiple post-translational modifications that regulate its activity, including phosphorylation and acetylation. Upon inflammatory stimulation, I*κ*B kinase (IKK) phosphorylates I*κ*B*α*, leading to its degradation and release of NF-*κ*B/p65 from I*κ*B*α*, which then undergoes nuclear translocation. Within the nucleus, p65 is acetylated by acetyltransferases such as p300/CBP at multiple lysine residues (e.g., K310), enhancing its DNA-binding affinity and transcriptional activity (47). SIRT1, an NAD^+^-dependent enzyme, directly deacetylates NF-*κ*B/p65, suppressing its transcriptional activity (37). In our experiments, NMN did not suppress the initial NF-*κ*B/p65 phosphorylation at 1 h after IL-1β stimulation (Supplementary Figure S3), indicating that NMN does not interfere with the upstream IKK-I*κ*B cascade. However, NMN significantly suppressed nuclear NF-*κ*B/p65 accumulation at 4 h and decreased expression levels of its target genes at 24 h (Figures 4C–G). This temporal pattern is consistent with a SIRT1-mediated deacetylation mechanism: NMN does not block the initial activation cascade, but promotes subsequent p65 deacetylation and inactivation, potentially leading to reduced transcriptional output and enhanced nuclear export. Supporting this mechanism, both a pharmacological SIRT1 inhibitor, EX527 (Figure 5), and genetic SIRT1 knockdown with siRNA (Figure 6) significantly abolished the protective effects of NMN, indicating that SIRT1 activity is required for the protective effects of NMN. Although direct assessment of p65 acetylation status would provide additional confirmation, our genetic, pharmacological, and functional evidence, combined with established literature demonstrating SIRT1-mediated p65 deacetylation (37), is consistent with this proposed mechanism in NMN-mediated endothelial protection. SIRT3, another NAD⁺-dependent deacetylase, also reduces nuclear NF-*κ*B/p65 levels in mouse models of inflammatory diseases and LPS-stimulated endothelial cells (48, 49). Therefore, both SIRT1 and SIRT3 may contribute to the suppression of the NF-*κ*B pathway by NMN in this study.

The NMN concentrations employed in this study (125–1,000 µM) exceed the plasma levels typically observed in human supplementation studies (50). However, several factors justify the physiological relevance of these concentrations. Previous studies have shown that a single oral administration of NMN (500 mg/kg) in mice increases tissue NAD⁺ levels approximately 1.2–2.3-fold depending on the organ (51). We compared intracellular NAD⁺ elevation rather than extracellular NMN concentrations, as tissue NAD⁺ dynamics—rather than plasma NMN levels—represent the functional endpoint most relevant to cellular biology. In the present study, treatment with 500 µM NMN increased intracellular NAD⁺ levels approximately 1.4-fold (Figure 5C). This degree of augmentation falls within the range reported *in vivo*, indicating that our *in vitro* system achieves a physiologically relevant magnitude of NAD⁺ elevation, despite the inherent differences between *in vitro* and *in vivo* pharmacokinetic environments. Furthermore, the correlation between NAD⁺-elevating capacity and protective efficacy across different precursors (NMNH > NMN = NR; Figures 5C,D) supports a mechanism-based effect linked to NAD⁺ metabolism instead of a nonspecific pharmacological effect of high extracellular NMN concentrations. Finally, NMN concentrations used in this study are widely used in *in vitro* studies investigating NAD⁺ metabolism and signaling pathways (24, 52, 53), as they enable measurable modulation of intracellular NAD⁺ pools under culture conditions and are considered standard for mechanistic studies in this field. Nonetheless, further pharmacokinetic analyses are required to more precisely define the relationship between *in vitro* exposure and tissue NMN dynamics *in vivo*.

The present study employed LPS-induced M1 macrophages as a model of pro-inflammatory activation. However, atherosclerotic plaques harbor considerably more heterogeneous macrophage populations, including triggering receptors expressed on myeloid cells 2 (TREM2)-high lipid-associated macrophages, Mox macrophages induced by oxidized LDL, M4 macrophages associated with plaque instability, and M(Hb) macrophages arising in areas of intraplaque hemorrhage, each contributing distinct mediators to the vascular microenvironment (54, 55). Within our experimental system, IL-1β neutralization markedly attenuated macrophage-conditioned medium-induced hyperpermeability, whereas TNF-α blockade had comparatively modest effects (Figure 2F), indicating that IL-1β plays a predominant role in these experimental conditions. However, coordinated cytokine signaling is widely recognized in endothelial activation; combined IL-1β and TNF-α inhibition has been shown to more effectively suppress HUVEC activation than either alone (27), supporting the view that these mediators act in concert to amplify endothelial barrier disruption. Beyond IL-1β and TNF-α, IL-6 has been shown to interact with TNF-α to exacerbate oxidative stress and impair eNOS phosphorylation, thereby contributing to coronary endothelial dysfunction in inflammatory disease models (56). Inflammatory vascular diseases involve complex immune cell interactions and multiple cytokine networks extending beyond the macrophage–endothelial axis examined here (57). Expanding future investigations to include diverse macrophage phenotypes and combinatorial cytokine stimulation, and evaluating NMN-mediated NAD⁺-SIRT1 protection in such settings, would better reflect the heterogeneous inflammatory environment characteristic of atherosclerosis and related vascular diseases.

Although the present study provides mechanistic insights into inflammation-induced endothelial dysfunction, several limitations should be carefully considered. First, our findings are derived from HUVECs rather than endothelial cells isolated from disease-relevant vascular beds. Although HUVECs are widely used for mechanistic studies of endothelial biology, endothelial heterogeneity across arterial, venous, and microvascular beds may influence quantitative responses. Future studies using arterial endothelial cells or other disease-relevant endothelial cell types are required to confirm the applicability of these findings in inflammatory vascular diseases. Second, our co-culture model was maintained under static conditions without hemodynamic shear stress. Shear stress is a critical regulator of endothelial phenotype, barrier integrity, and inflammatory signaling *in vivo*. Future studies incorporating flow-based systems would enable more physiologically relevant evaluation of NMN-mediated endothelial protection under inflammatory conditions. Finally, further validation in disease-relevant models and *in vivo* systems is required to determine the applicability of these findings beyond the current experimental setting.

In conclusion, this study investigated the protective effects of NMN on vascular endothelial cells against M1 macrophage-derived IL-1β-induced hyperpermeability. Using a 3D co-culture model of HUVECs and M1 macrophages, we revealed that NMN attenuated IL-1β-induced endothelial hyperpermeability by inhibiting VE-cadherin degradation and intercellular gap formation in an NAD^+^-SIRT1-dependent manner. These findings provide a rationale for further investigation of NMN in inflammatory vascular diseases.

## Data Availability

The original contributions presented in the study are included in the article/Supplementary Material, further inquiries can be directed to the corresponding author/s.
