# Supplementary material for: NMN protects vascular endothelial cells from M1 macrophage-derived IL-1β-induced hyperpermeability by inhibiting VE-cadherin degradation
Source: Front Cardiovasc Med. 2026 May 18;13:1748872. doi: 10.3389/fcvm.2026.1748872 (PMC13222840; doi:10.3389/fcvm.2026.1748872)
Supplement: Supplementary file 1 [file Datasheet1.pdf]

S1

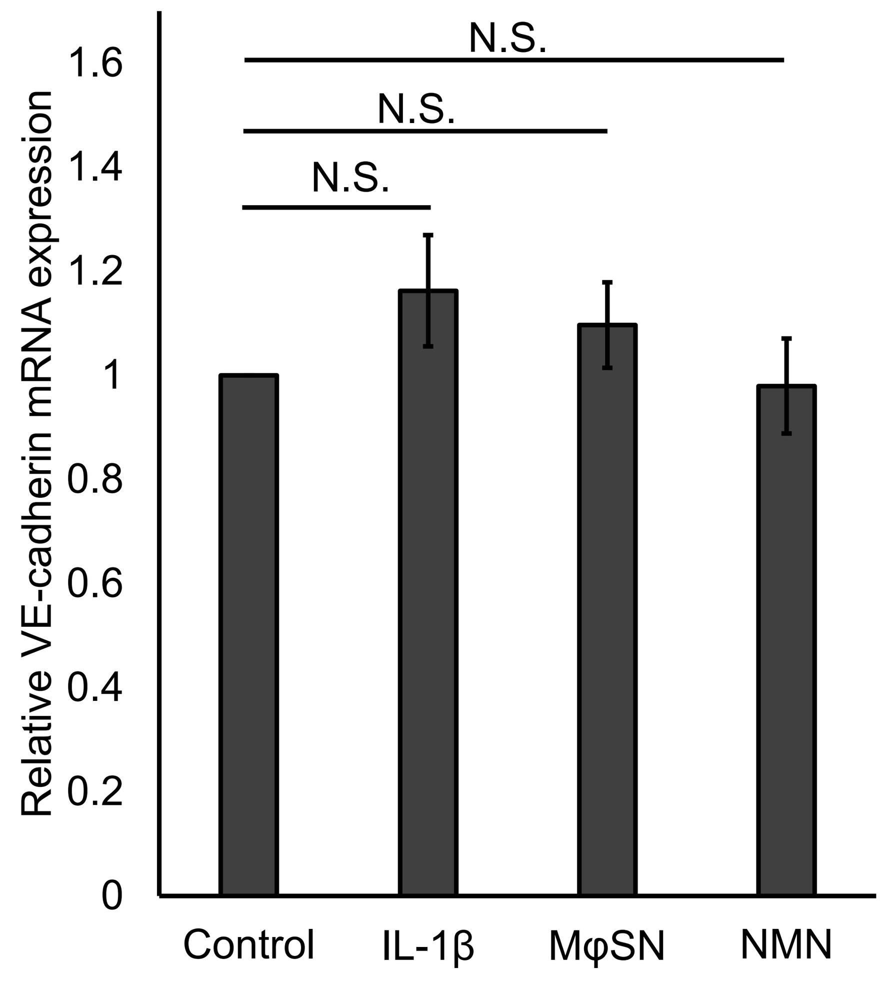

S2

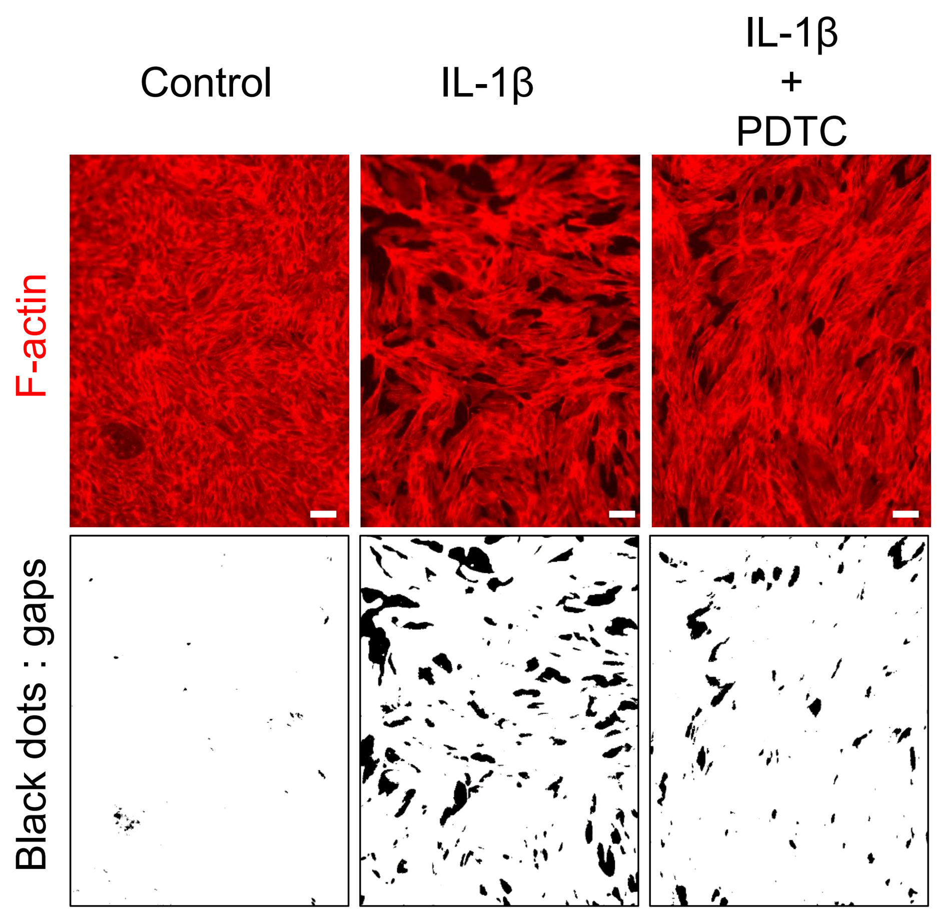

S3

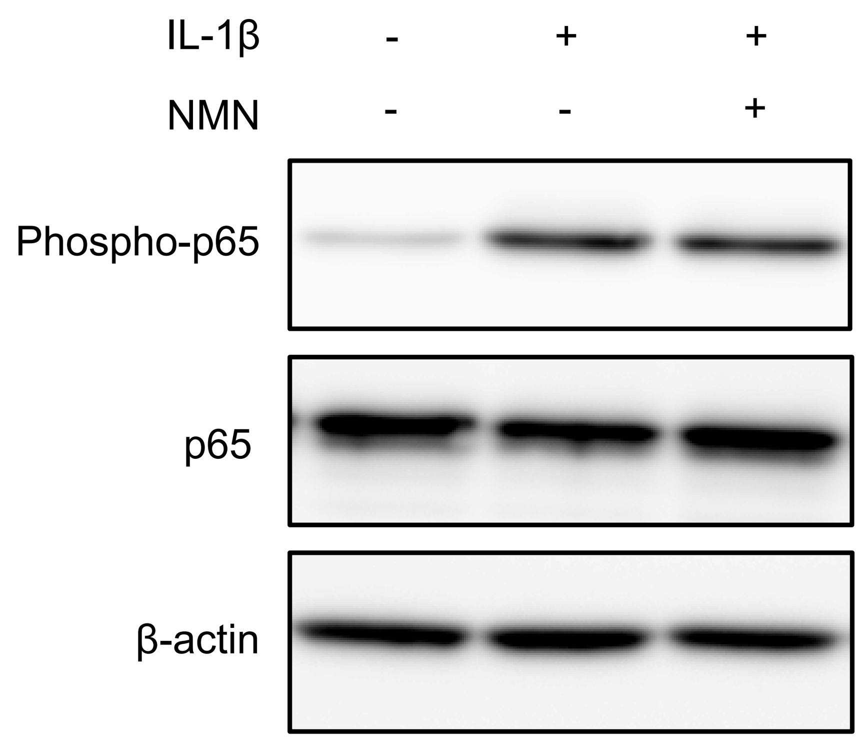

S4 A **B**

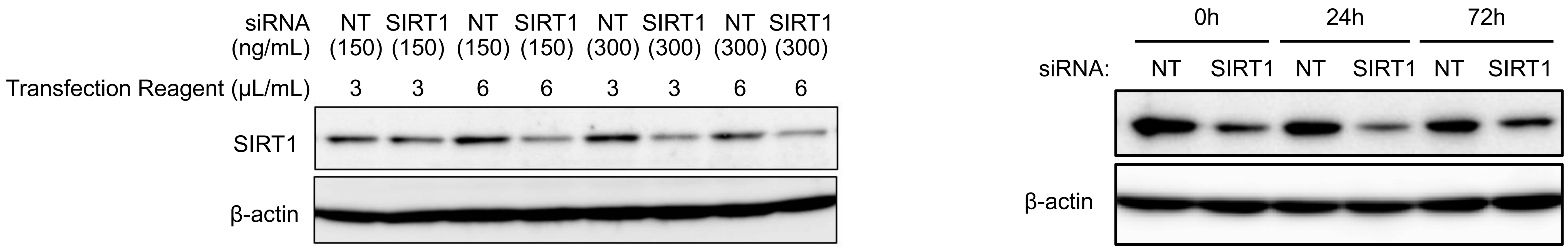

Supplementary Figure 1. HUVECs seeded in 12-well plates were incubated with 1 ng/mL IL-1β, M1 macrophage culture supernatant (MΦSN), or 500 μM NMN for 24 h. Thereafter their VE-cadherin mRNA expression levels were determined using qPCR. N.S.: not significant. Data are presented as mean ± SEM; n = 3 independent experiments, each performed in triplicate.

Supplementary Figure 2. HUVECs plated onto cell culture inserts were pretreated with 100 μM NF-κB inhibitor (PDTC) for 30 min and incubated with 1 ng/mL IL-1β for 48 h. The F-actin cytoskeleton was stained with Alexa Fluor 594 phalloidin (red). Captured images were binarized using a global threshold. Black dots in the adjusted images represent intercellular gaps in the HUVEC monolayer. Intracellular gap areas were measured as the ratio of the black dot area to the entire image using ImageJ software. Scale bar: 100 μm. Representative immunofluorescence images are shown. Data are presented as mean ± SEM; n = 3 independent experiments.

Supplementary Figure 3. HUVECs seeded in 12-well plates were pretreated with 500 μM NMN for 24 h and incubated with 1 ng/mL IL-1β for 60 min. Phosphorylation levels of NF-κB/p65 were evaluated using western blotting with an anti-phospho-p65 and anti-p65 antibodies. β-actin served as a loading control. Representative blots are shown. Data are presented as mean ± SEM; n = 3 independent experiments.

Supplementary Figure 4. (**A**) HUVECs were transfected with Nontarget (NT) or SIRT1 siRNA (150-300 ng/mL) using 3-6 μL/mL HiPerFect Transfection Reagent for 24 h. (**B**) The medium was then replaced with growth medium, and the cells were incubated for 0–72 h. Intracellular SIRT1 levels were evaluated using western blotting with an anti-SIRT1 antibody. β-actin served as a loading control. Representative blots are shown. Data are presented as mean ± SEM; n = 3 independent experiments (B).

Figure 3C. Original western blot

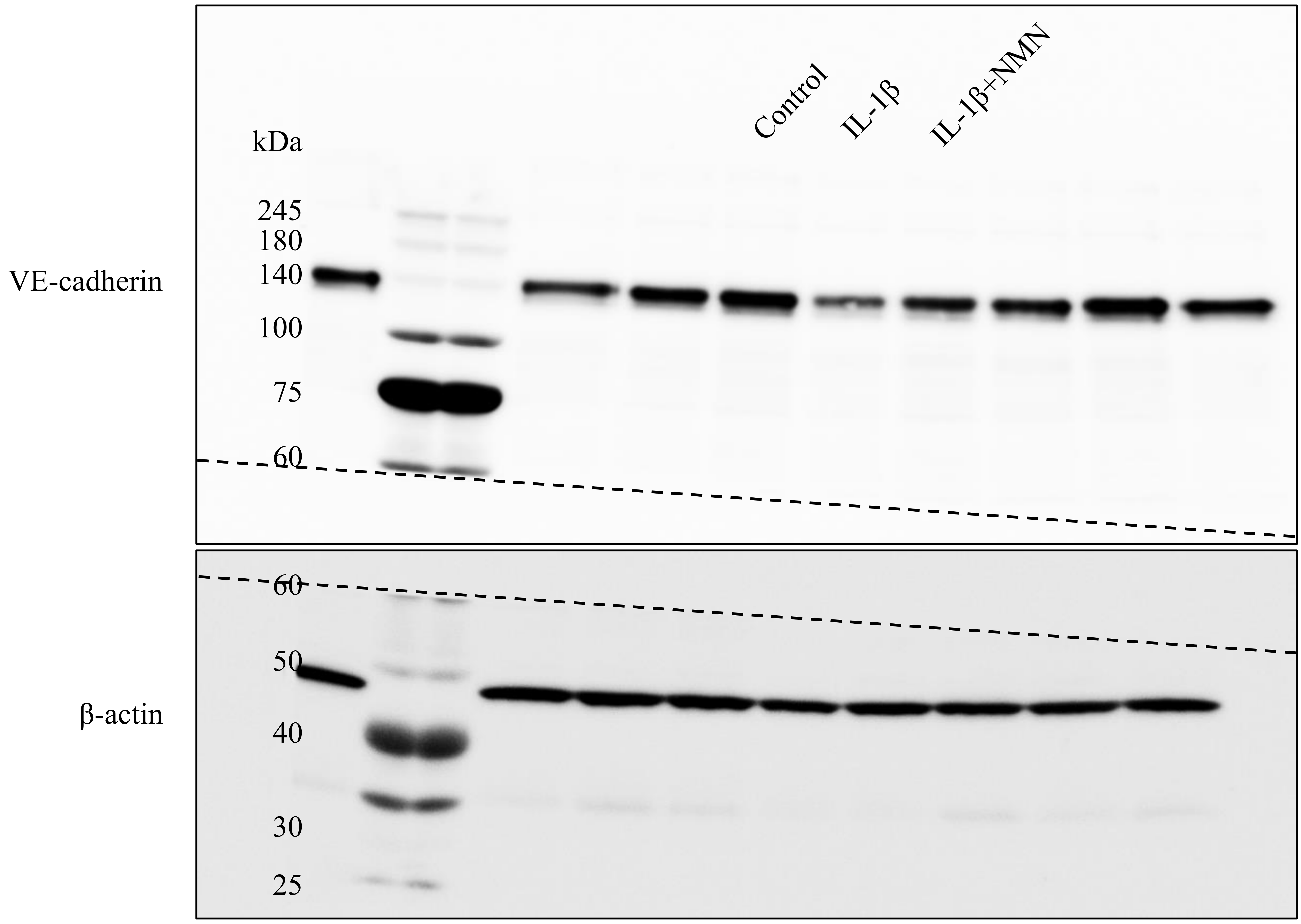

The dotted line indicates where the membrane was cut.

Figure 4B. Original western blot

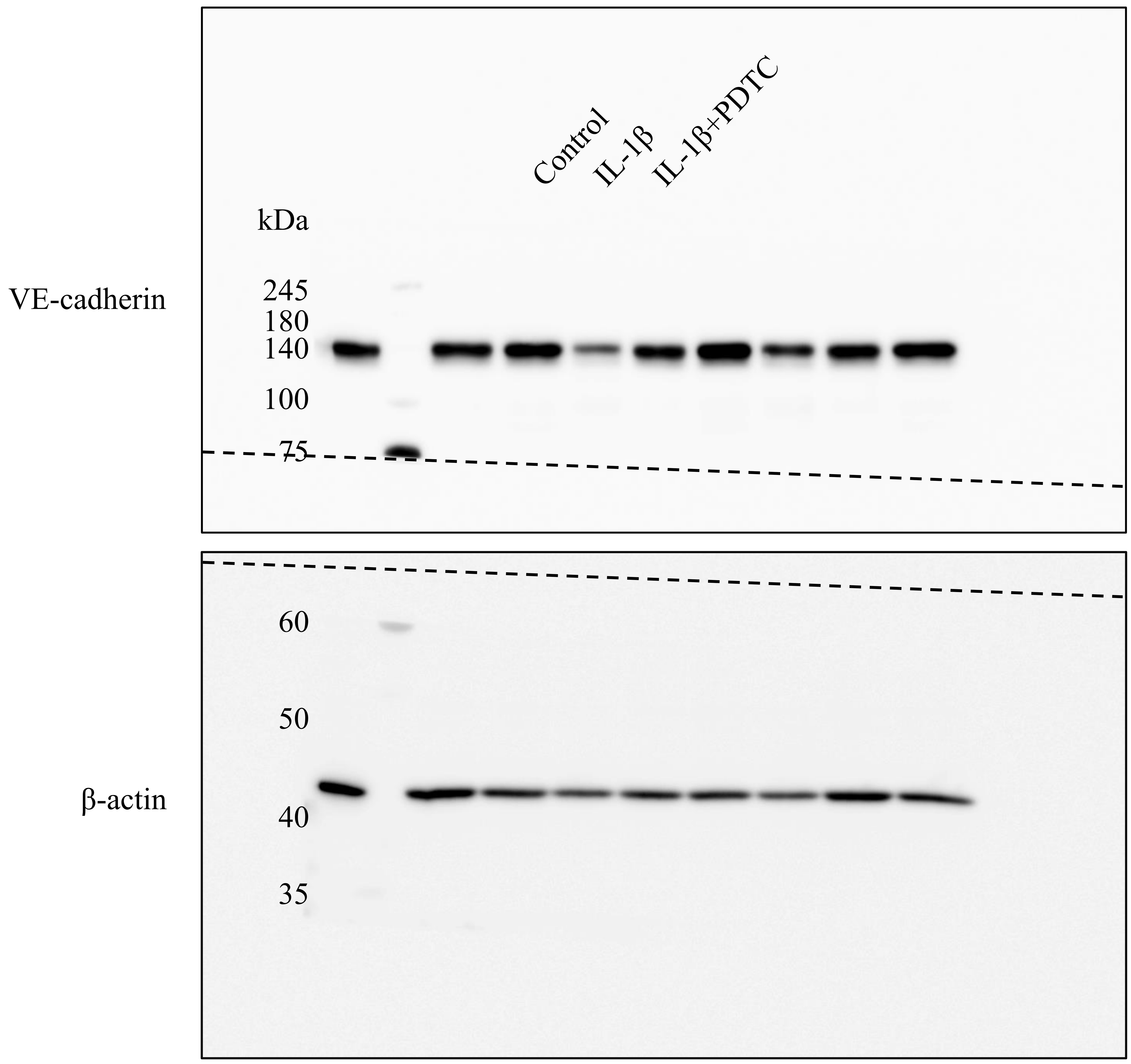

The dotted line indicates where the membrane was cut.

Figure 5B. Original western blot

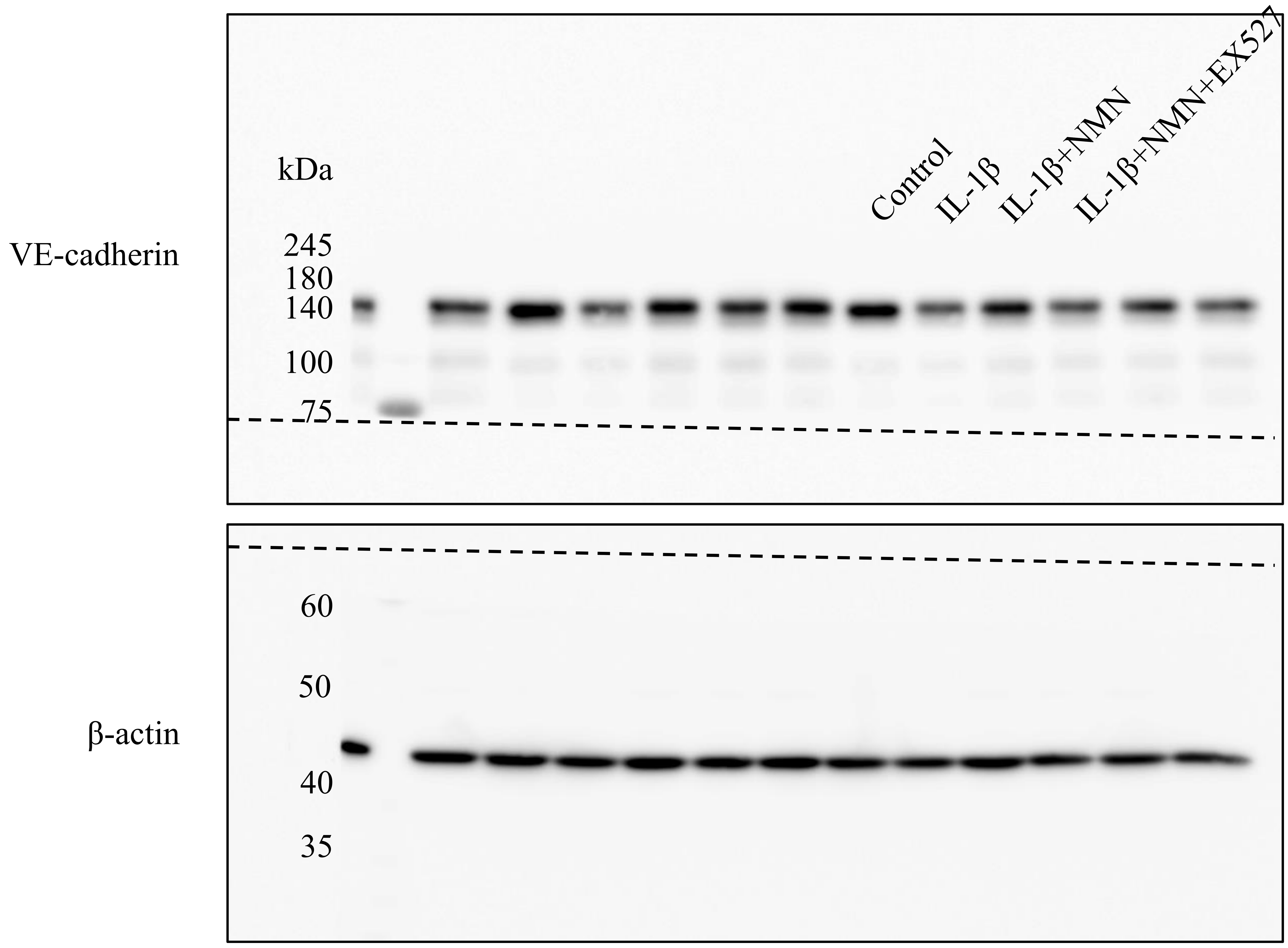

The dotted line indicates where the membrane was cut.

Figure 6B. Original western blot

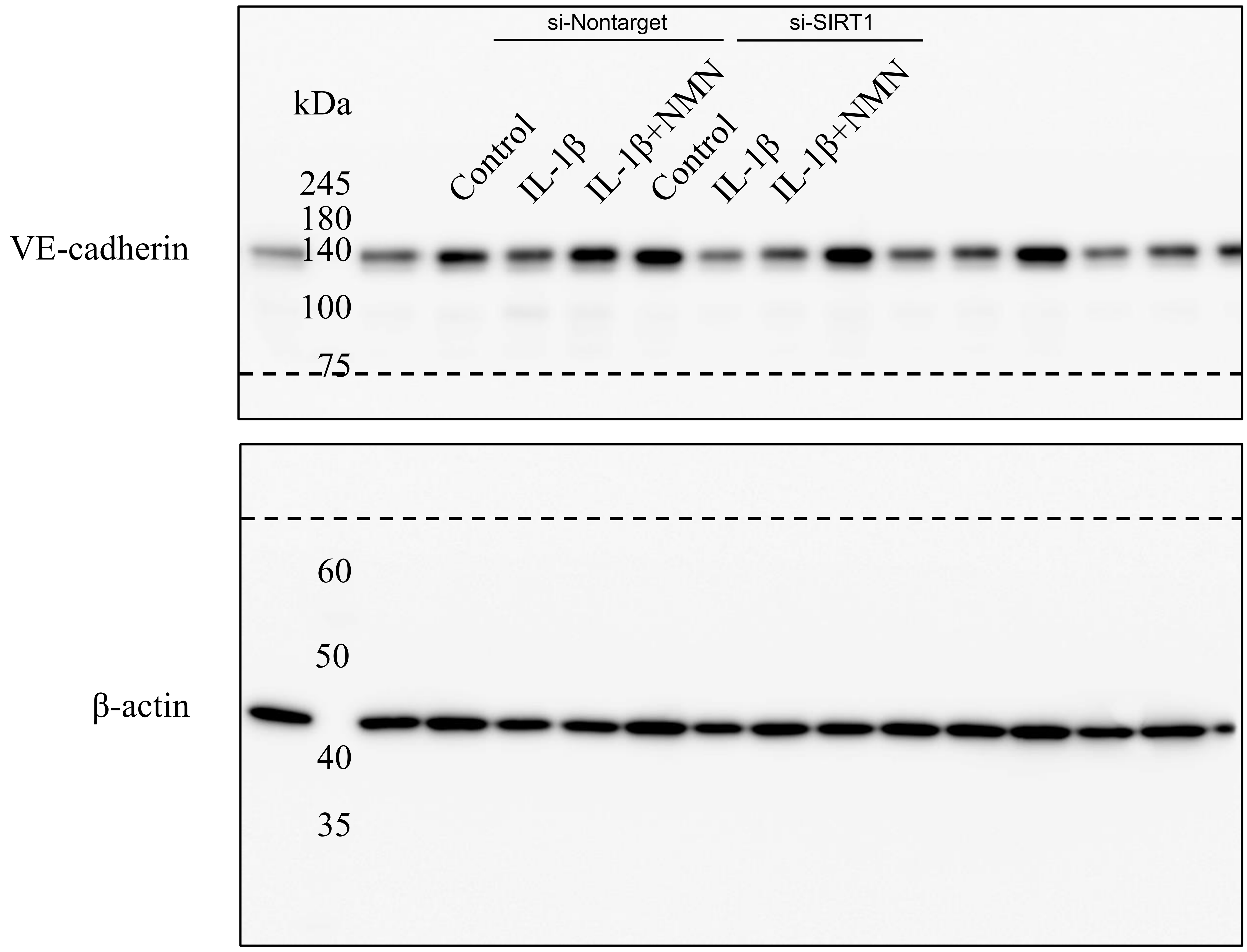

The dotted line indicates where the membrane was cut.
